# Emergent mechanisms of evidence integration in recurrent neural networks

**DOI:** 10.1371/journal.pone.0205676

**Published:** 2018-10-16

**Authors:** Silvan Quax, Marcel van Gerven

**Affiliations:** Radboud University, Donders Institute for Brain, Cognition and Behaviour, Nijmegen, the Netherlands; Universiteit Gent, BELGIUM

## Abstract

Recent advances in machine learning have enabled neural networks to solve tasks humans typically perform. These networks offer an exciting new tool for neuroscience that can give us insight in the emergence of neural and behavioral mechanisms. A big gap remains though between the very deep neural networks that have risen in popularity and outperformed many existing shallow networks in the field of computer vision and the highly recurrently connected human brain. This trend towards ever-deeper architectures raises the question why the brain has not developed such an architecture. Besides wiring constraints we argue that the brain operates under different circumstances when performing object recognition, being confronted with noisy and ambiguous sensory input. The role of time in the process of object recognition is investigated, showing that a recurrent network trained through reinforcement learning is able to learn the amount of time needed to arrive at an accurate estimate of the stimulus and develops behavioral and neural mechanisms similar to those found in the human and non-human primate literature.

## Introduction

Recent developments in neural networks offer a promising new avenue for studying the mechanisms of the brain. Neural networks can be used to gain insight across many different scales of processing, from single neuron activity to populations of neurons and entire cortical areas. More importantly, they have now evolved to a point where they can learn to perform a very broad variety of complex tasks on similar performance levels as humans. Exploring the mechanisms that a neural network develops to solve a particular task can give us valuable insight into the basic ingredients needed for certain complex behaviors to arise. This adds an exciting new tool to neuroscience research that can help us to solve questions of ‘how’ and ‘why’ a certain mechanism develops, moving beyond the comparison of purely descriptive models of neural and/or behavioral phenomena.

For these neural networks to become a useful tool for studying the brain there is still a big gap to bridge. On the one hand we have to develop the building blocks of these networks in such a way that a useful comparison with the brain can be made. On the other hand the circumstances in which the networks learn a particular task should be more representative of typical circumstances encountered in the natural world, i.e. under certain levels of noise and ambiguity or limited time to make a decision.

Computer vision researchers are adopting ever-deeper neural networks to solve complex, human-like tasks such as the recognition of natural objects [1]. While these networks are very successful at solving these tasks, their success comes at the cost of needing more and more computational units (artificial neurons) to create these deep layers. The very deep residual networks used in current object recognition tasks are nearly equivalent to a recurrent neural network unfolding over time, when the weights between their hidden layers are clamped [2]. Recurrent networks may come close to the performance of deep residual networks, while using substantially fewer parameters.

When considering the human visual cortex, we could equate layers in a neural network with the different processing stages in visual areas of the ventral stream, where every area represents a nonlinear transformation of the previous area. The number of subsequent areas or processing steps in the human visual cortex are typically thought to be between 7–9 to move from sensory input up to inferior temporal cortex (IT) [3], where object recognition is thought to take place. This is an order two of magnitude less than the depth of residual networks, that are typically in the hundreds of layers, but can have over a thousand [1].

One reason for the relatively low number of processing stages in human cortex could be the transmission delays between neurons in the different areas. These are typically on the order of 10 ms and would impair an organism with very long reaction times when similar depths as residual networks would be used. The human brain is able to perform object recognition in approximately 150 ms [4]. Evolution could have pushed the brain towards efficient transformations between cortical areas, such that correctly recognizing one’s environment can be achieved in a few computational steps.

Another important aspect is the number of neurons and synapses that are needed by a neural network. Since the brain is limited by spatial and metabolic constraints, it is important to minimize the number of neurons and synapses as much as possible, by using recurrent computations. Local recurrent connections and long-distance feedback are thought to play an important role in the efficient processing of information. Evidence shows that recurrent computations in the brain play an important role in a wide variety of tasks involved in visual perception [5].

When considering efficient information processing, the nature of the information itself is a crucial factor that determines the best neural architecture to process this information. Our natural environment tends to be highly temporally correlated, and has causal relationships that can have a wide range of time spans [6]. This means that we do not need to completely infer our environment from scratch at every moment in time. Instead, we can make use of these temporal dependencies to reduce the number of inferences we need to make in order to arrive at a good estimate of the state of our environment. Information reaching our sensory neurons tends to be noisy, full of ambiguity and incomplete. This makes it essential for the brain to integrate sensory evidence over a certain period of time, before drawing conclusions about the underlying causes for the sensory evidence. Humans and non-human primates tend to be very efficient in integrating sensory evidence over time [7]. That the brain uses time to resolve noise in sensory input, is reflected in the longer reaction times that subjects have when more noise is present in their stimuli [8]. Interestingly, there can be a trade-off between the speed of responses and the accuracy, depending on the rewards or penalties that a task presents [9].

Recent advances in machine learning have enabled neural networks to solve tasks humans typically perform. The question remains whether the mechanism by which they solve a particular task is similar as in humans and whether the behavior and neural activity of the network match those found in human and primate literature. This grants us insight into which assumptions and components used for the neural network are necessary and sufficient for the emergence of human like behavior and neural mechanisms. In the present paper, we explore these questions by using a perceptual decision making task that is often investigated in humans and non-human primates to study the role of time in perceptual decision making. We study how a recurrent neural network can integrate noisy sensory information. Through supervised learning we train the model to perform optimally for different integration times. However, the brain usually has no access to such supervised labels. Moreover, supervised learning does not provide the flexibility of deciding when to stop integrating information. Therefore, we show that reinforcement learning enables the model to make efficient use of time, while trying to reach optimal performance, effectively learning the integration time from data.

We compare the behavior and neuronal responses of the network with findings from the human and non-human primate literature to see whether our network develops similar mechanisms as found in humans and non-human primates. This allows us to identify whether the basic ingredients of our network are sufficient for the emergence of the neural mechanisms that underly complex human behavior.

## Results

### Integrating evidence over time

**Fig 1:**
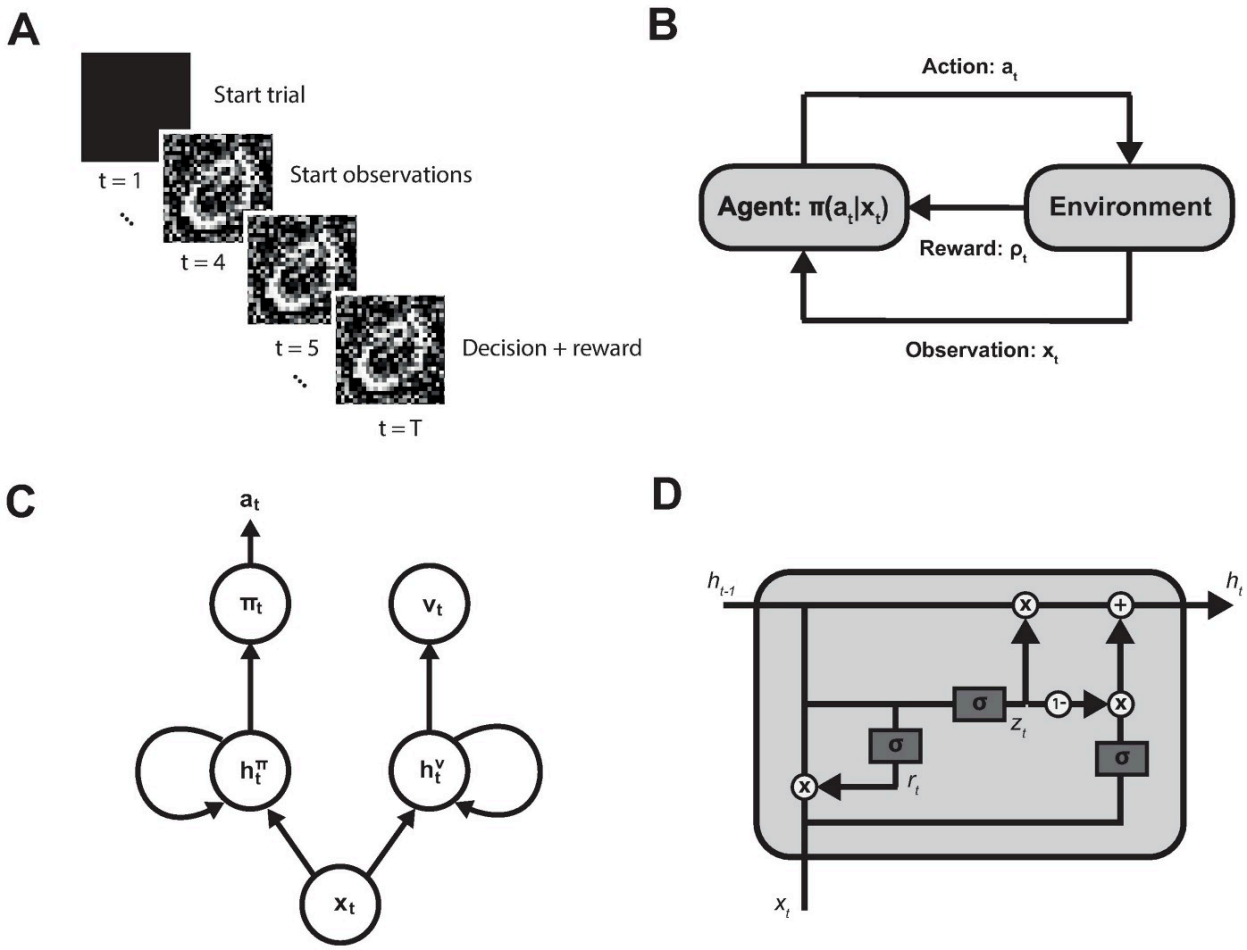
Overview of the model. **(A)** The task consisted of trials during which noisy stimuli from the MNIST dataset were shown. A trial started with a black screen. After three time steps the noisy stimulus appeared. In the supervised case the trial length was predetermined, while in the reward based learning case the agent could decide at every time step to either make an extra observation or choose one of the stimuli classes. Once one of the classes is chosen a trial ended. **(B)** In the reward based learning experiments a reinforcement learning agent is trained. The agent receives observations *x*_*t*_ and rewards *ρ*_*t*_ from the environment at every time step. Based on the learned policy *π*_*t*_(*a*_*t*_|*x*_*t*_) the agent chooses one of three actions *a*_*t*_ (extra observation, class 1 or class 2). **(C)** Our reinforcement learning agent consisted of a neural network trained through an actor-critic algorithm. There were two recurrent networks, one making up the actor part that returned the policy and one making up the critic part that returned a value representing the expected future reward. **(D)** The hidden units of the recurrent network were made up of Gated Recurrent Units, that contain two internal gating units *r*_*t*_ and *z*_*t*_ that gate how the input information *x*_*t*_ and internal hidden unit activity *h*_*t*−1_ is combined for the hidden activity in next time step *h*_*t*_.

To see how a recurrent neural network deals with perceptual decision-making under noisy conditions, we used an object recognition task. The network was trained using multiple trials, where each trial consisted of a pre-stimulus period, during which a black screen was shown and a period after stimulus onset during which the stimuli were shown. An overview of the task is shown in Fig 1A. The trial length was either fixed, in the supervised case, or flexibly determined by the choices made in the reward-based case. In the reward-based case the model learned through an actor-critic reinforcement learning algorithm based on observations and rewards it receives from the environment. A detailed description of the learning algorithms can be found in the Methods section. Fig 1B shows a schematic overview of the agents interaction with the environment. The agent itself is made up of a neural network that consists of two parts, one encoding the policy that determines the action chosen and one encoding the expected value of future rewards. A comprehensive overview of this network is shown in Fig 1C The input observation at every time step is sent to both the policy and value network where it is combined with the internal hidden activity of the network. The hidden units combine this information through a Gated Recurrent Unit (GRU) [10], that is shown in Fig 1D. The mathematical details of these GRU’s are given in Eqs 1–3 of the Methods section.

The MNIST [11] dataset was used to train the network to classify handwritten digits. Noise was added as described in Methods and Materials. To see how a network can integrate information over time we started with a network trained through supervised learning, where two different noise conditions were being tested. The first ‘static noise mask’ condition, maintained the same noise mask during an entire trial, thus leading to the same information being presented to the network at every time step. The second ‘dynamic noise mask’ condition maintained the same noise level during an entire trial, but had a noise mask that changed at every time step, thus leading to different information being presented to the network at every time step. We trained networks of different temporal lengths, such that a networks loss function was only based on the classification performance at the last time step.

**Fig 2:**
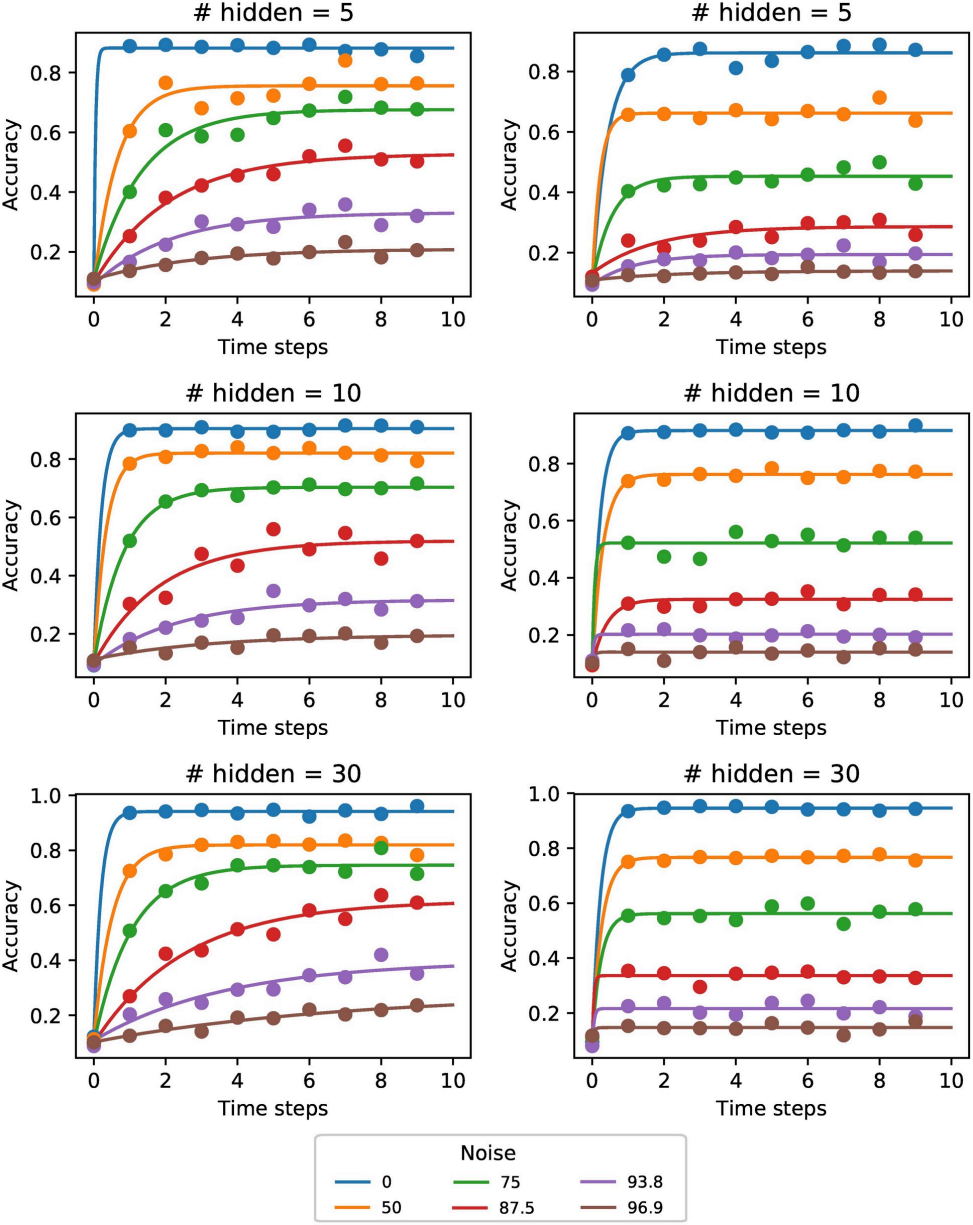
Classification accuracy over time. Classification accuracy of supervised network under different noise levels and for different network sizes (number of hidden units). Dynamic noise (left) vs static noise (right). With dynamic noise we observe an increase in accuracy with the number of time steps the network has to classify the stimulus. For higher noise levels the network requires a longer integration time before the maximal performance is achieved.

Fig 2 (right) shows that in the static noise condition, more time steps were not leading to better classification performance. Even under more noisy conditions no benefit was observed from adding extra time steps. We compared this with the case where a different noise mask was generated at every time step. In this case more time steps were very beneficial (Fig 2, left), indicating that the network was integrating information over time. For higher noise levels there was more benefit from integrating information over time.

**Fig 3:**
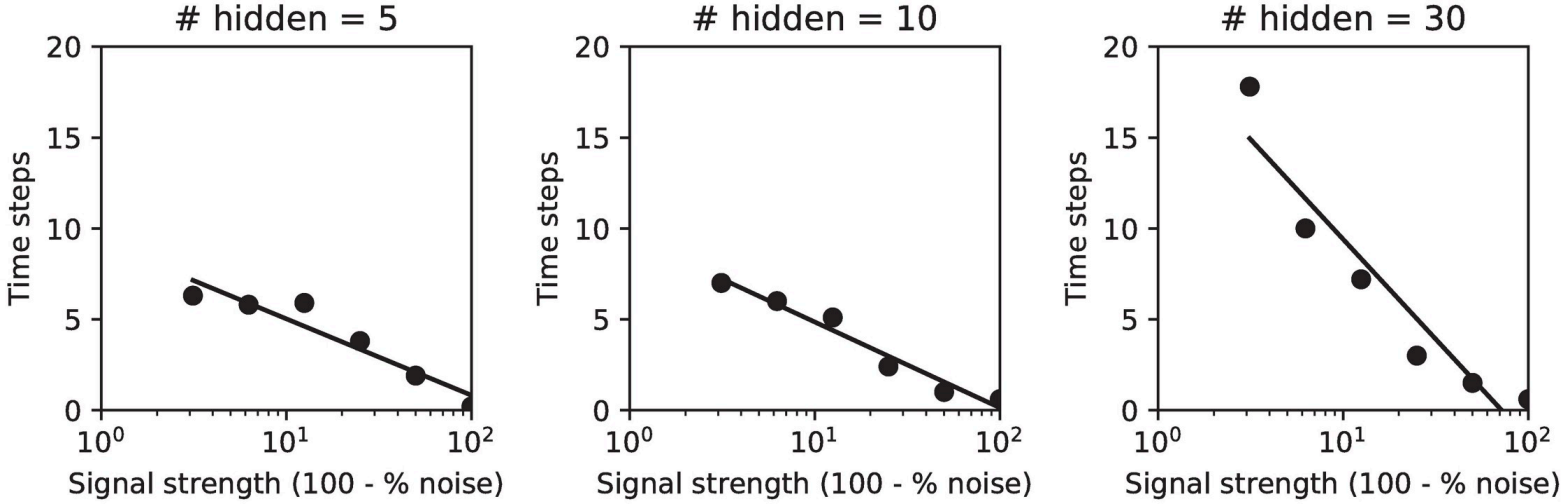
Time needed for accurate decoding. The number of time steps needed to reach 90% of its maximum performance in the dynamic noise condition as a function of the signal strength (i.e. 100—% noise). For lower signal strength (i.e. higher noise levels) it takes more time steps for the network to reach 90% of its maximum performance. Fewer hidden units lead to less capacity to improve performance over multiple time steps.

To determine how many time steps the supervised agent needed to reach a good classification performance in the dynamic noise condition, we examined the number of time steps it took the network to reach 90% of its maximum classification performance (Fig 3). The measure of signal strength, which is just the opposite of noise (i.e. 100—% noise), is used for easier comparison with experimental results. For lower signal strength (i.e. higher noise levels) it took more time steps to reach the maximum performance.

To see how the number of hidden units of the network influenced integration over time, we trained three different networks with 5, 10 and 30 hidden units. A smaller capacity of the network might force it to make use of more time steps. On the other hand, more hidden units might lead to more complex recurrent dynamics that could benefit from integration over time. Overall the larger network of 30 hidden units benefited most from extra time steps (Fig 3C), indicating that the higher capacity allows more complex dynamics that help with integrating evidence.

### Learning the number of observations needed for classification

Since the brain often has limited time and resources before a perceptual decision is made, it has to trade off accuracy for speed. In a natural environment there is a need for adaptive behavior in order to perform efficient classification of incoming noisy stimuli. While some stimuli can be classified very rapidly with a high certainty, others need a considerable amount of time to integrate information over before a choice can be made. We investigated whether we can train such an efficient learner to choose its observations adaptively depending on the incoming stimulus. To this end, we trained an actor-critic model using reinforcement learning as described above and detailed in the Materials and Methods section. These actor-critic policy gradient based algorithms have recently become popular in the field of machine learning by being able to learn to solve complex reinforcement learning tasks such as Atari games [12]. Some evidence seems to indicate that the brain might act according to a similar mechanism. Increases in the blood-oxygen-level dependent (BOLD) responses of the ventral striatum have been linked to representations of expected future reward, thus performing a similar function as the critic. At the same time, the dorsal striatum shows increased BOLD responses only when a subject has to act according to the expected future reward, thus performing a role similar to the actor [13].

We investigated whether this network was able to learn how many observations are needed to achieve optimal performance rather than imposing a particular temporal depth. We trained the network to decide between two classes of MNIST or request an extra observation at the current time step. The network was trained using different levels of dynamic noise, since only in this condition a benefit for extra time steps was found during supervised training.

**Fig 4:**
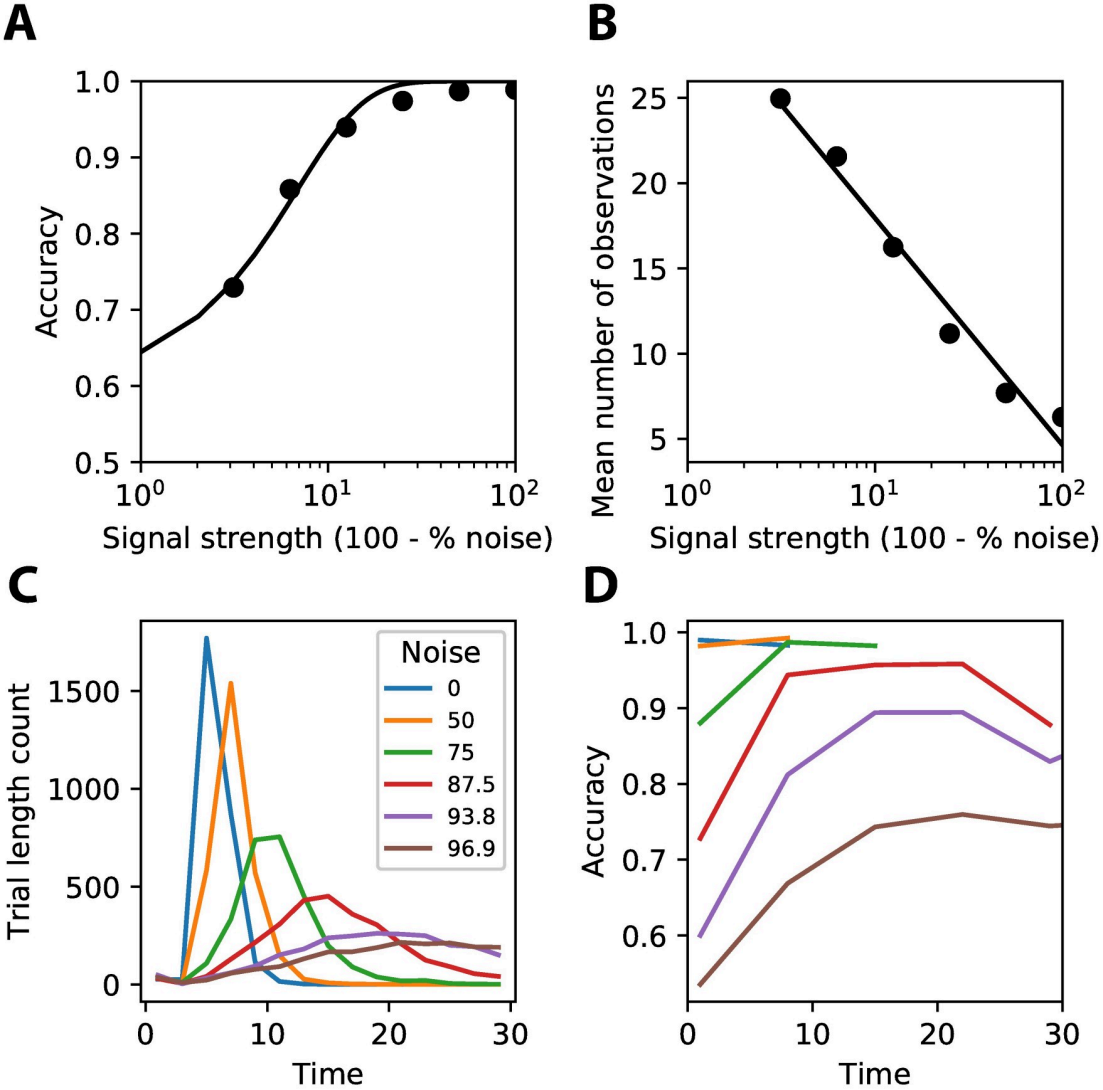
Behavior of trained reinforcement learning agent. **(A)** The accuracy that the agent achieves at different levels of signal strength (i.e. 100—% noise). Less signal (i.e. more noise) makes it harder for the agent to classify correctly (solid line represents fit of psychophysical function). **(B)** The average number of observations that the agent uses before making a decision on the class. More observations are made before deciding for trials with lower signal strength (i.e. higher noise levels) (solid line represents linear fit). **(C)** The distribution over trial lengths for different noise levels shows that higher noise leads to longer reaction times, but also to more variance in the reaction times.

Fig 4A shows the classification accuracy of a trained network for these different levels of signal strength. The network was able to achieve a high accuracy for all levels of signal strength, with performance only dropping slightly for the lowest levels of signal strength (i.e. highest noise levels). Due to the interaction of exploration and exploitation of the reinforcement learning agent the performance achieved during different training sessions could vary (S1 Fig). Exploitation of a certain policy might prevent the agent from learning the best possible solution.

The network learned to make efficient use of time to achieve good performance. For stimuli with less signal strength (i.e. more noise), more observations were made before one or the other class was chosen (see Fig 4B). The network was thus able to learn to adjust the number of observations needed to the level of signal strength of the incoming stimuli. The fact that the network is not always using the entire length of the trial to integrate information is induced by the discounting of future reward (*γ*, Eq 5), which ensures that the network prefers getting the same reward now rather than in the future, thus driving the network to decide early if no gain in reward is expected anymore. Discounting of future rewards has also been observed in human and animal studies that have suggested a role for dopamine neurons encoding this information [14, 15]. Fig 4C shows the histogram of the number of trials with a certain amount of time steps for the different noise levels. The trial length for low noise stimuli is short and sharply peaked, while for higher noise levels trials are much longer and there is more variability. Recall that a single network was trained on the different noise levels, without explicit information on the amount of noise in a given trial. The network was thus able to infer the amount of noise from its observations and adjust its behavior accordingly. Similar behavior is found in macaques on perceptual decision making tasks with different amounts of apparent motion coherence (compare Fig 4A and 4B with Fig 3 of [8]).

**Fig 5:**
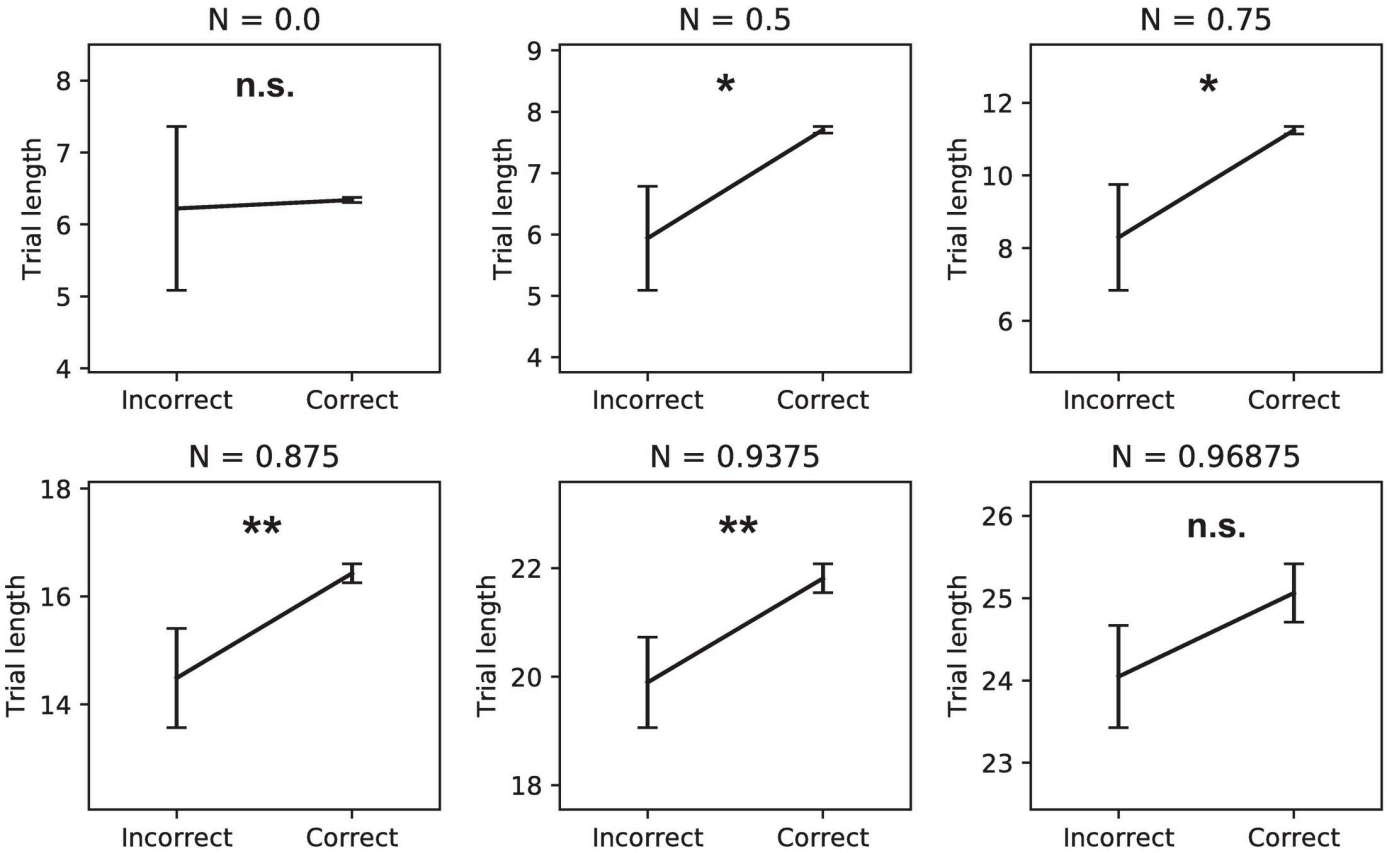
Time benefits performance. Correct trials used more time steps than incorrect trials. This effect is only visible when noise is added to the stimuli, because there more benefit is to be gained from extra observations. At the highest noise level there might be a cut-off effect because trials could not exceed 50 observations (* *p* < 0.0001, ** *p* < 0.01).

We analyzed the average trial lengths for incorrect versus correct responses for all noise levels, to examine whether longer trial lengths really benefited performance (Fig 5). Trial lengths were on average longer for correct responses than incorrect responses, showing the benefit of making extra observations. Interestingly, the experimental literature shows that, depending on the experimental manipulation, different patterns of reaction times on incorrect versus correct trials can emerge. A similar pattern as observed in our model is seen when subjects are pressed to respond fast, for example by having a penalty when a response is not given fast enough. This task setup is very similar to the penalty of not getting any reward if no response is given in time in our setup, which could explain the similarity between the behavior of our model and these studies [16].

**Fig 6:**
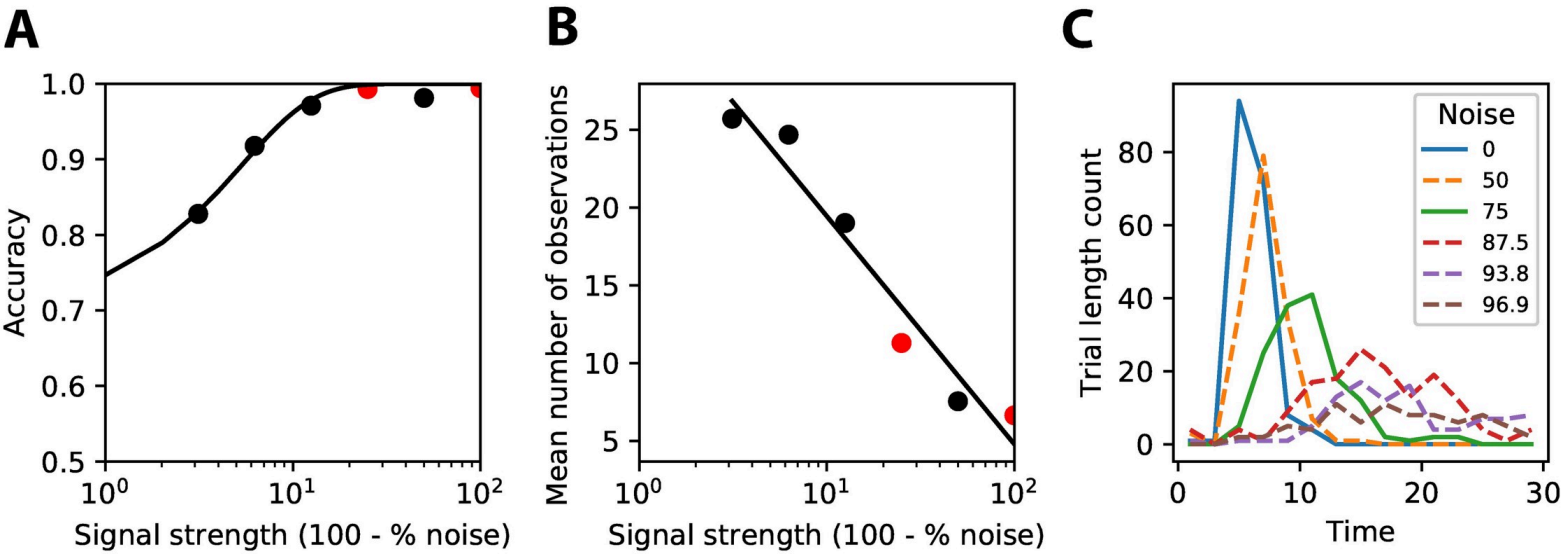
Learned behavior generalizes to different noise levels. The network was trained on two noise levels (0% and 75%, red dots). During testing all 6 noise levels were shown **(A)** The agent achieves good performance on noise levels that were not seen during training (black dots). **(B,C)** The agent also learns to use an adequate number of observations on noise levels not seen during training (black dots **(A)**, dashed lines **(C)**), indicating it has really learned a mechanism to integrate information and make a decision when enough evidence is collected.

Generalization to conditions that have not been observed before is an important feature of a neural mechanism, since it enables an agent to learn and adapt quicker to its environment, without having to extensively train on all possible variations in which a stimulus can be observed. To see whether our network really learned a general mechanism for perceptual decision making and not just a separate strategy for every noise level, we tested whether the network was able to generalize to previously unseen viewing conditions (noise levels). We trained a network on just two noise levels (0% and 75%), while showing all levels of noise during testing. The network is able to both interpolate and extrapolate to unseen noise levels (Fig 6), while learning the same mechanism even when shown just two levels of noise.

**Fig 7:**
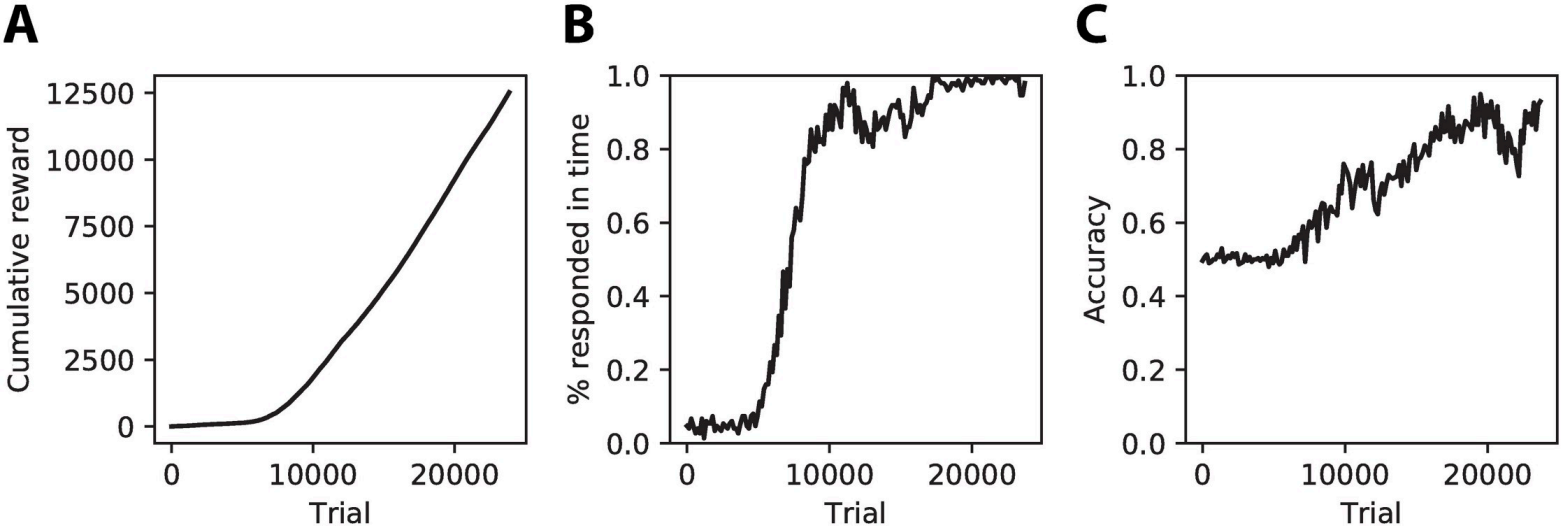
Learning behavior of the reinforcement learning agent. **(A)** The cumulative reward that the agent has obtained starts increasing after approximately 7000 trials. **(B)** This coincides with the moment that the agent learns to choose one of the classes within the time limits of a trial. **(C)** The classification accuracy of the agent also starts to increase at this point, slowly reaching a good level of performance.

The learning behavior of the agent showed that initially it is unable to choose the actions that lead to reward, since the total accumulated reward remains very low (Fig 7A). The agent learns at some point to choose one of the classes within the time limits of the trial as shown by the sharp increase in the percentage of trials where one of the classes is chosen within the time limits of a trial (Fig 7B). From this point the classification accuracy starts to increase slowly until it reaches good performance (Fig 7C).

### Neural activity of the network

To see whether the network learned to solve the perceptual decision making task through a similar neural mechanism as found in humans and non-human primates, we analyzed the activity of the neurons in the network.

**Fig 8:**
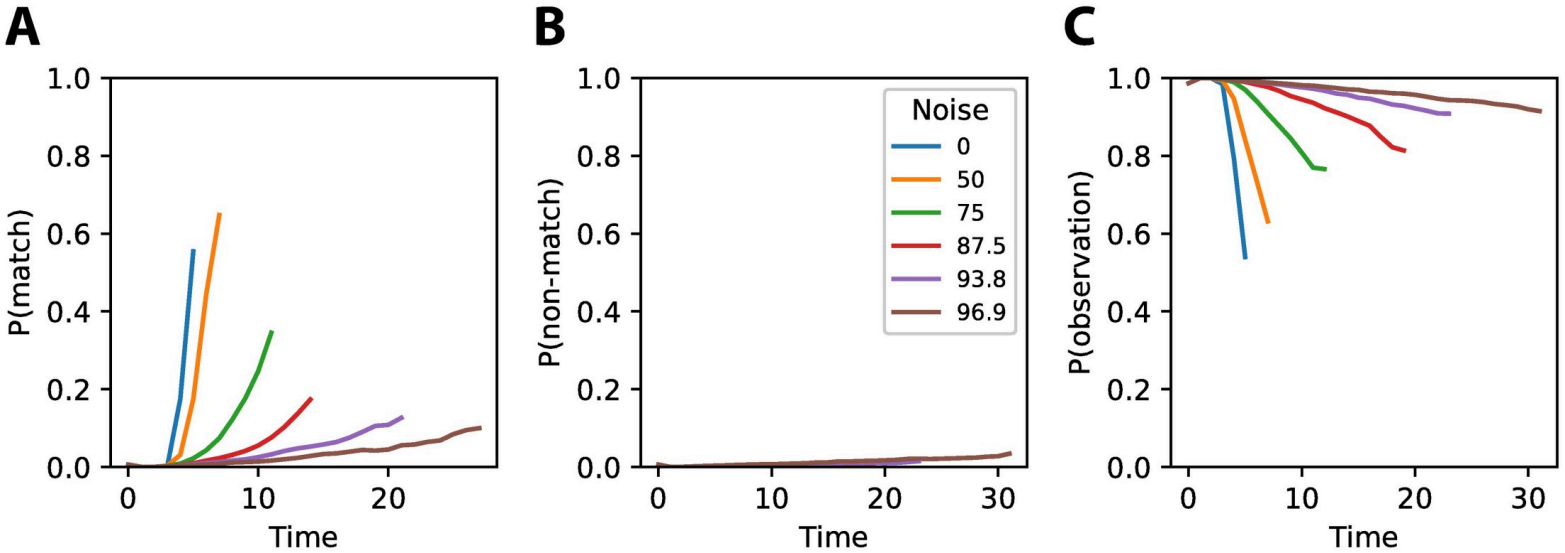
Neural activity of the output neurons. **(A)** Activity of the output unit on trials where the stimulus matching the output unit was shown. Activity increases over time representing a higher confidence in the respective stimulus class. For lower noise levels activity increases faster, indicating that evidence is accumulated faster. **(B)** Activity of the output unit on trials where the stimulus not matching the output unit was shown. This activity is kept low during the entire trial and for all noise levels, indicating that very little evidence is collected for the non-matching class. **(C)** Activity of the output unit corresponding to making an extra observation.

The activity of the output unit matching with the presented stimuli class increased over time (Fig 8A). Less noise led to a faster accumulation of evidence, leading to an earlier decision for the correct class. On the other hand did the probability of the output unit not matching the presented stimulus class not increase, the chance of choosing the wrong class was kept to a minimum (Fig 8B). The output unit representing the probability of taking an extra observation starts high and decreases over time depending on the noise level of the observed stimulus (Fig 8C), ensuring that a decision is delayed until sufficient evidence is collected.

**Fig 9:**
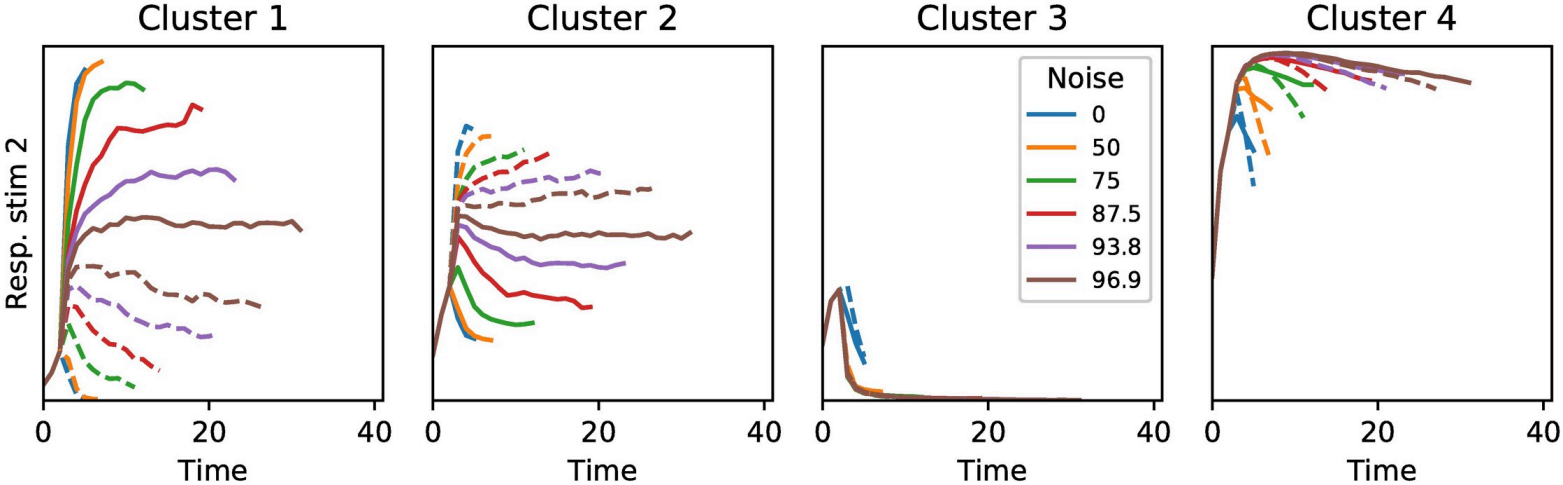
Clustered activity of the hidden neurons. The activity of the hidden units in the network could be clustered into four distinct clusters. The responses for the different clusters are shown for stimuli of category one (solid line) and stimuli from category two (dashed line). Colors represent the different noise levels. Cluster 1 increases its activity when there is more evidence for stimuli from category one, while Cluster 2 increases its activity when there is more evidence for stimuli from category two. Cluster 3 strongly decreases its activity after the fixation period during which no response should be given, thus indicating when evidence integration starts and responses can be given. Cluster 4 seems to increase its firing rate if the noise level is high, independent of the specific stimulus category that is shown. These could function as an extra representation of uncertainty.

To understand how the hidden units of the network gave rise to this behavior, we analyzed the activity of the hidden units during a trial. We used k-means clustering as described in the Methods and Materials section to find common response patterns among the hidden units. The activity could be clustered into four distinct clusters (Fig 9). Two clusters of neurons, Cluster 1 and 2, encoded the amount of evidence for respectively stimulus class one and stimulus class two. These units increased the activity when their respective class was presented, while they decreased activity if the other class was shown. The rate at which their activity increased or decreased depended on the amount of noise in the observations. These neural activities are remarkably similar to neural recordings performed on macaque monkeys, where neurons in LIP have also been found to increase or decrease their activity depending on whether a stimulus matches a receptive field, and where the rate of activity increase or decrease depends on the amount of noise in the stimulus, with more noisy stimuli showing a slower increase or decrease in firing rate (compare Fig 9, Cluster 1 and 2 with Fig 7A of [8]). The third cluster of neurons has learned to indicate when the fixation period is finished and responses lead to rewards. The fourth cluster of neurons is indifferent to the stimuli class that is presented, but does seem to encode the amount of noise that is present in the observations. This information could be used to determine whether to the request an extra observation, which is also supported by the fact that the activity for this cluster of neurons looks remarkably similar to the output probability of an extra observation as shown in Fig 8C.

### Trading off speed for accuracy

**Fig 10:**
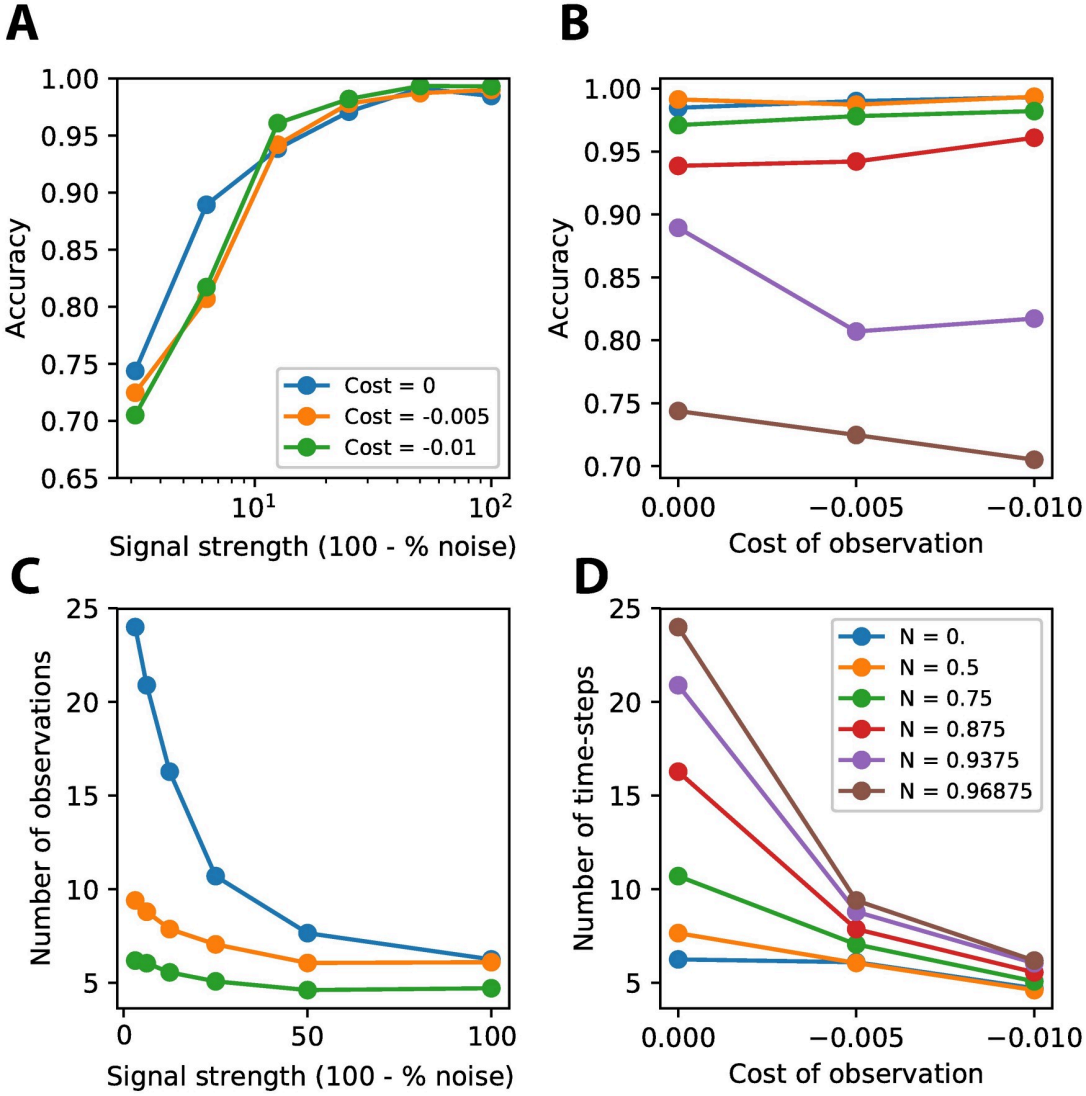
Speed accuracy trade-off. The network is able to respond faster, but less accurate when a cost is attached to extra observations. The accuracy only decreases slightly on lower signal strength levels (higher noise levels) due to this speed-accuracy trade-off **(A,B)**. The number of observations decreases for different costs imposed to extra observations **(C)**. The strongest decrease is seen for lower signal strength levels (higher noise levels) **(D)**.

Humans are able to trade off the speed of their responses for their accuracy. When higher costs are imposed on long reaction times, humans tend to respond faster, but more inaccurately [9]. To see whether our network is able to learn similar trade-offs, we trained our agent under three conditions. The cost imposed on extra observations was set to either 0, −0.005 or −0.01 per observation. There was a trade-off in accuracy only for stimuli with low signal strength (i.e. very noisy stimuli) (Fig 10A and 10B). The network learned to respond faster when higher costs were imposed for extra observations (Fig 10C). The trials with low signal strength (i.e. noisy stimuli) that showed a decrease in accuracy also showed the largest decrease in the number of observations (Fig 10D). These results agree well with the behavior found in macaque monkeys performing a task where they had to trade-off speed and accuracy (compare Fig 10A and 10C with Fig 2A of [17]).

## Discussion

Our network shows behavior that is similar to how humans act on comparable tasks of perceptual decision-making [8]. We show that this network is able to learn actions that ensure efficient gathering of information. This mechanism is able to generalize to viewing conditions that have not been observed during training, showing the robustness of the emerging mechanism. Furthermore we show that internal activations of the neurons in the network are comparable to neural responses found in the non-human primate literature. A trade-off between speed and accuracy can be accomplished be assigning negative rewards to extra time steps in the network. The emerging behavior resembles findings from human and macaque experiments on the trade-off between speed and accuracy [9, 17].

Interestingly, our networks that were trained on stimuli with static noise masks, which did not change over observations, showed no improvement when processing information over time. From experimental literature it is clear though that humans do take longer processing time on noisy images with a static mask [18]. A possible cause for this discrepancy can come from the fact that our model, as opposed to humans, has no internal noise that can be reduced by longer integration times. An interesting avenue for future research would be to incorporate internal noise in the model and see what the effect of internal versus external noise is on the integration of information. Some recent findings show that convolutional recurrent networks improve their performance when an image is processed for multiple time-steps, both on images without noise [2] and images with a static noise mask [19]. This difference could come from the use of convolutions in these networks instead of densely connected layers. A possible reason that convolutional recurrent networks do show such an effect could come from the fact that convolutions only see a small patch of pixels at a time, where using multiple time steps could lead to integrating information over multiple of these patches.

Although it is a major advancement that our networks were able to deal with actual images as stimuli, it remains a question how our model holds for stimuli that are even more complex, such as high resolution natural images or videos. With higher complexity, a single layer fully connected network is probably not be enough, and multiple layers with convolutional computations could become necessary for good performance. Recent work has achieved good performance on the classification of natural images with these convolutional recurrent neural networks [20].

There are still challenges to be overcome in the training of recurrent neural networks. Vanishing and exploding gradients have been a problem for recurrent neural networks for a long time [21]. A surge in solutions for this problem [22] have contributed to the recent success of recurrent neural networks in a wide variety of applications [23]. Still the success of the training depends strongly on the choice of hyperparameters, which are often chosen by trial and error, or based on the experience of the experimenter. A more formal understanding of what makes these networks behave well, and when they fail to train would greatly benefit the use of neural networks in neuroscience.

Recent work has highlighted how reward-based training of neural networks can solve tasks that are often performed in studies involving humans and non-human primates [24–26]. Behaviour of these networks is remarkably similar to how humans perform these tasks, as also substantiated by our findings. These networks present a promising tool for discovering complex neuronal mechanisms that underly human behaviour. The simplicity of training a general neural network for a certain task, compared to hand engineering a model, has proven useful for finding common mechanisms that serve a role in the execution of a wide range of tasks [26]. Most reinforcement learning algorithms for neural networks rely on stochastic policies. Often this stochasticity is important for exploration to occur and not end up in some local minimum. How realistic this stochasticity is in the context of the brain remains to be seen though. One observation we made is that the activity in our neurons do not drift to a certain threshold that deterministically decides if a action is chosen or not. While evidence for such a threshold in the brain exists [8], it is impossible to model it with stochastic policies. Since a stochastic policy always chooses actions according to a certain probability, a slow drift of the output probabilities toward a threshold would not ensure that the correct action is chosen at the exact moment the threshold is reached, but could just as well be chosen before or after reaching the threshold depending on the probabilistic action selection. Further work on deterministic policies [27] could provide a closer link to the brain in the future.

While formal comparison of our networks policy with other methods is hard to achieve due to its stochastic nature, there are behavioral traits that have also been found in previous methods. The increasing trial lengths for noisy stimuli during training agree with previous findings of increasing decision boundaries during learning in a deferred-decision making task [28]. Recently there has also been evidence of decision boundaries decreasing over time in a trial can be the most optimal policy [29]. Our output probabilities when a decision is made for the correct class are lower for longer high noise trials versus the short low noise trials. This could be an indication that our network is decreasing its decision boundary during a trial if it has difficulty gathering enough evidence for one of the classes. Especially since there is a penalty if a trial takes too long, it would be a wise strategy. We cannot exclude though that this lower decision probability is due to the stochasticity of the policy, that might lead to a choice if there are multiple low probability time steps in a row. Similarly there has also been evidence that humans and animals increase the cost they associate with taking extra observations during a trial [30]. This could lead to similar behavior as decreasing decision boundaries, functioning as an urgency mechanism. In a future, more formal, experimental setup this method would be very interesting to uncover the internal costs that our network has learned to associate with extra time steps.

An important issue still remaining in learning cognitive tasks through reinforcement learning is the problem of meta-learning, or the generalization of certain skills over task conditions and between different tasks. Humans have the remarkable ability to extend previously learned skills to a new context very fast, while neural networks often require complete retraining. Our network was able to generalize along the noise dimension of our task, showing similar properties as recently found by [31], who showed for example how a network was able to learn a strategy for optimizing reward in an armed bandit task that generalized over reward probabilities not seen during training. The problem of generalization to completely new tasks that only partly share commonalities is a much harder problem though and one of the biggest challenges currently facing our field.

The training of recurrent neural networks can be regarded as a tool to achieve a certain network configuration that is able to solve particular cognitive tasks. For this purpose, supervised learning can be a valid option to achieve fast and stable solutions. If the learning procedure and development of the network are also subject of study, unsupervised learning or reinforcement learning may be more realistic models of how the brain learns. While these learning paradigms still face many challenges, important advances have been made recently [32, 33]. While most networks still depend on biologically implausible versions of the backpropagation algorithm for the updating of their weights, there are recent attempts to devise more biologically plausible learning rules [25, 34–36].

In bringing neural networks closer to human information processing, we hope to reveal those components and mechanisms of the human brain that are essential for its functioning. Determining which mechanisms are essential for which brain function, and which are mere epiphenomena that are just by-products of our evolutionary development will be greatly benefited by having these computational models. Instead of performing complex and costly lesion studies, we can switch on and off parts of a network by a mere press of a button. Neural networks are still far removed from being biological plausible models of the brain though. The development of deep neural networks has enabled us to perform complex tasks on natural stimuli, but to use neural networks to understand the brain they should be limited by similar constraints as the brain. Choosing parameters like connectivity and temporal delays that are based on physiological data should improve the explanatory power of neural networks for neuroscience data. By validating that emergent properties of the networks, like firing rates and dynamics are similar to observations from the brain can help us to strengthen our beliefs that mechanistic effects found in these networks can also apply to the brain.

Vice versa by developing these networks to learn under more natural conditions of noisy or ambiguous over time, and learning the adaptive behavior that our network shows will benefit applications in more natural environments. Especially practical applications that require online processing of data like, for example, self-driving cars will have to deal with trading-off the speed of their processing with the accuracy of their inferences. When neural networks are applied in natural environments, noise and ambiguity is unavoidable and time will be of much greater importance than in the classification of static images. Important mechanisms for integrating evidence over time, non-classical receptive field effects and using temporal correlations to constrain inference could benefit the expressiveness of these recurrent neural networks. Less computational depth, requiring fewer hidden units, will help keeping the computational resources needed within bounds when performing online object recognition. [37]. Reinforcement learning mechanisms as shown in this work will be essential to achieve adaptive and flexible inference in these applications.

In future research biological plausibility can be improved be restraining the parameter regime to biologically relevant values, while also moving towards stimuli that even closer represent our natural environment. This not only means using natural images, but actual temporal correlated streams of natural input, preferable in interaction with an agent’s actions. Such improvements of biological plausibility of these recurrent neural networks and the conditions in which they are trained will enable us to directly identify components of our model in the brain. Correlating model responses and neuronal data of subjects performing the same task can then reveal in detail which neural populations are involved in a certain cognitive task, and whether they work according to similar mechanisms as developed in the recurrent neural network model.

We have shown how a mechanism of perceptual decision making can emerge in a neural population when confronted with noisy stimuli, through reinforcement learning. The importance of time in the process of object recognition when confronted with noisy and ambiguous sensory input is demonstrated, showing that a recurrent network trained through reinforcement learning is able to learn the amount of time needed to arrive at an accurate estimate of the stimulus. Finally the behavioral and neural mechanisms that the network develops have been shown to be similar to those found in the human and non-human primate literature. This demonstrates how neural networks can be a useful tool to understand the right conditions and requirements necessary for the development of neural mechanisms and opens up an exciting path for future research to further our understanding of neural mechanisms essential to human cognition.

## Materials and methods

### Task setup

To see how a recurrent neural network deals with perceptual decision-making under noisy conditions, we used an object recognition task. The network was trained using multiple trials, where each trial consisted of a pre-stimulus period, during which a black screen was shown and a period after stimulus onset. The trial length was either fixed, in the supervised case, or flexibly determined by the choices made in the reward-based case.

**Fig 11:**
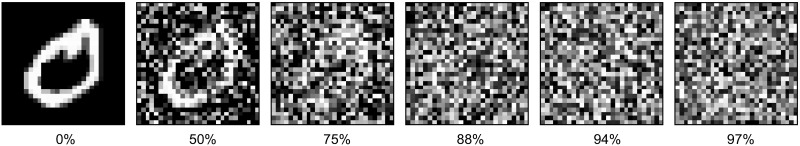
Stimuli used in simulations. Example MNIST stimuli at different noise levels, from left to right: 0%, 50%, 75%, 88%, 94%, 97%. In a single trial, a stimulus and a noise level were randomly selected. At every time step a new observation was created by generating a new noise pattern.

As stimuli we used Modified National Institute of Standards and Technology (MNIST) images [11] with variable amounts of noise. Noise was introduced by replacing different proportions of pixels (0%, 50%, 75%, 88%, 94%, 97%) with random values from a uniform distribution between 0 and 1. Example stimuli for the different noise levels are shown in Fig 11. All ten image classes were used for the supervised experiment, while a subset of two classes was used for the two-alternative forced choice (2AFC) task in the reinforcement learning (RL) experiment.

In the case of supervised learning a fixed read-out time was chosen and the network was trained to minimize the loss between this classification and the correct label as described in the Supervised Learning section below.

During the reward-based simulations, the agent received rewards based on the choices made and should learn to maximize its reward. The agent could choose from three actions during the whole trial. That is: get an extra observation, choose class 1 or choose class 2. As soon as one of both classes were chosen the trial ended. Choosing the extra observation led to an extra time step with an extra observation of the stimulus.

### Reward structure

The agent was able to obtain different rewards during a trial. Choosing one of both stimulus classes before stimulus onset led to a reward of 0, since there was no right answer to be given yet. Choosing the right class after stimulus onset led to a reward of +1, while choosing the wrong class led a reward of 0. If no class was chosen after 50 time steps a reward of 0 was given and the trial ended.

### Network architecture

We used a two-layer recurrent neural network to learn the task, with the first layer converting input to hidden activities and a second layer converting hidden activities to output. For the first set of experiments training was done in a supervised way using a softmax loss function. The second set of experiments used an actor-critic reinforcement learning (RL) algorithm based on the REINFORCE algorithm [38] with an added value function, where both the policy and value function were learned by our network. All training was done using the Chainer toolbox [39] for Python.

All neural networks that were used were two-layer recurrent neural networks, with the first layer consisting of a variant of gated recurrent units (GRU) [10] converting input **x** to hidden activations **h** and a second, dense layer, converting hidden activations to output **z**. The GRU unit is able to exploit two kinds of sigmoidal gates that modulate the influence of the hidden activations at the previous time step on the current hidden activations according to the following equations:

$$\begin{array}{ccc} r_{t} & = & \sigma (U_{r}x_{t}+W_{r}h_{t-1}+b_{r}) \end{array}$$ (1)

$$\begin{array}{ccc} z_{t} & = & \sigma (U_{z}x_{t}+W_{z}h_{t-1}+b_{z}) \end{array}$$ (2)

$$\begin{array}{ccc} h_{t} & = & \tau z_{t}⊙h_{t-1}+\left(1-\tau z_{t}\right)⊙\sigma \left(U_{z}x_{t}+W_{h}\left(r_{t}⊙h_{t-1}\right)+b_{h}\right) \end{array}$$ (3)

where *σ*(⋅) is the sigmoid function applied to each of the arguments and ⊙ is the Hadamard product. Vectors **r** and **z** are both functioning as gates for the activity of the hidden units **h**. *U* and *W* are weight matrices weighing the contribution from the input and the previous hidden state respectively. The bias is denoted by **b**. These parameters are learned during the training of the network. We added a constant *τ* = 0.5 that regulates the temporal profile of the dynamics. We used a sigmoid activation function for the hidden units **h**_*t*_ which ensures that activities are always positive and thus comparable to firing rates of real neurons. In case of the supervised experiments, the output consisted of the image classes. In case of the RL experiments, the output consisted of a policy and a value, as described later on and illustrated in Fig 1C.

### Supervised learning

In the first part of our study we trained networks through supervised learning. The network was trained to classify the input stimuli on the final time step of the recurrent neural network as belonging to one of *K* possible classes. Different readout times were tested, i.e. 1–10 steps. The network was trained by backpropagation through time (BPTT) [40], which allows weight updates of the network to be influenced by past input to the network. We used a softmax cross-entropy loss function where

$$\begin{array}{c} \zeta =-\sum_{k=1}^{K}t_{k}\cdot \log y_{k} \end{array}$$ (4)

with $y_{k}=e^{z_{k}}/\sum_{l=1}^{K}e^{z_{l}}$ the softmax output for the *k*-th class and *t*_*k*_ the target output. Minimizing this loss function ensures that the softmax of the network outputs converges to the desired target values.

### Reward-based learning

In a reinforcement learning setting an agent has to learn to choose actions, *a*_*t*_, that can lead the agent at every time point either to rewards or costs, *ρ*_*t*_. The agent selects actions from a policy, *π*_*t*_, by chance according to the respective probabilities corresponding to every action. The goal of the agent is to find those actions that maximize the expected future reward

$$\begin{array}{c} J=E[\sum_{t=0}^{T}\gamma \rho_{t}] \end{array}$$ (5)

where *γ* is the reward discounting factor and the expectation is taken over all possible trajectories. Since we cannot simulate every possible trajectory, we approximate it by averaging over a finite number of trials.

The objective function we aim to minimize is given by

$$\begin{array}{c} Q=\frac{1}{N}\sum_{n=1}^{N}[-J_{n}+0.5\cdot V_{n}+R_{n}^{\pi }] \end{array}$$ (6)

with *N* the total number of trials, *J*_*n*_ the reward for a given trial, *V*_*n*_ the value baseline and $R_{n}^{\pi }$ a regularization term given by the entropy of the policy [41]. The REINFORCE algorithm can be used to minimize this objective function [38]. This policy gradient algorithm requires the calculation of the gradient of this objective function with respect to its parameters. To further improve convergence, we added a value network to incorporate a baseline, which leads to the advantage actor-critic algorithm [12]. This baseline value should give an estimate of the expected future reward that the actor can get in its current state.

The advantage actor-critic algorithm uses the first term of Eq (6), defined as

$$\begin{array}{c} \nabla J_{n}=\sum_{t=0}^{T}\nabla \log\pi (a_{t}|x_{t})A_{n} \end{array}$$ (7)

where the policy for action, *a*_*t*_, given observation, *x*_*t*_, is represented by *π*(*a*_*t*_|*x*_*t*_) and the advantage term is given by

$$\begin{array}{c} A_{n}=\sum_{t^{\prime }=t}^{T}[\rho_{t^{\prime }}-v\left(x_{t^{\prime }}\right)] \end{array}$$ (8)

the advantage term, which gives the discrepancy between expected rewards, *v*, for observation, *x*_*t*′_, and real rewards, *ρ*_*t*′_. As such, this enhances actions that lead to higher than expected reward, while decreasing the probability of actions leading to lower than expected reward.

The value baseline we used is given by

$$\begin{array}{c} V_{n}=\frac{1}{T+1}\sum_{t=0}^{T}{\left[\sum_{t^{\prime }=t}^{T}[\rho_{t^{\prime }}-v\left(x_{t^{\prime }}\right)]\right]}^{2} \end{array}$$ (9)

which is the sum of the advantage squared at every time step. Minimizing this part ensures that expected rewards approach truly obtained rewards.

The regularization term is based on the entropy of the policy and serves to bias the agent towards more exploration

$$\begin{array}{c} R_{n}=\sum_{n=1}^{N}\pi \left(a_{n}|x\right)\log\pi \left(a_{n}|x\right) \end{array}$$ (10)

where *N* is the number of possible actions the agent can choose. This is achieved by pushing action probabilities away from 0 and 1, such that the agent does not get stuck in choosing the same action over and over.

Both the policy *π* and the value *v* were given by separate neural networks, as described in the Network Architecture section above, that converted the input image through a series of non-linear functions. Both networks consisted of 100 hidden units. Training lasted for 200000 time steps, which could lead to a varying number of trials depending on the choices made by the agent.

### Analysis

#### Fitting behavioral accuracy

In order to quantify how much the classification accuracy of the network increased with the number of time steps that were taken, we fit an exponential function to the data:

$$\begin{array}{c} f(t)=A\exp(Bt)+C \end{array}$$ (11)

with three free parameters *A*, *B* and *C*. We were mainly interested in the growth parameter *B*, as this gives us a measure of the increase in accuracy from one time step to another. The exponential was fitted using the non-linear least squares method implemented by the curve_fit function from Python library Scipy [42].

#### Clustering of responses

For the clustering of hidden unit activities the KMeans function from the Scikit Learn [43] library for Python was used. The k-means method uses two steps to find clusters, C, of activity patterns, **X**. First the data vectors are assigned by minimizing the sum squared distance

$$\begin{array}{c} d=\sum_{k=0}^{K}\sum_{x\in C_{k}}||x-\mu_{k}{\left|\right|}^{2} \end{array}$$ (12)

of every data point, **x**_*j*_ ∈ **X** to the mean of their assigned cluster. Second, the cluster centroids, ***μ***_*k*_, are recalculated such that they move to the center of the new cluster

$$\begin{array}{c} \mu_{k}=\frac{1}{|C_{k}|}\sum_{x\in C_{k}}x\;. \end{array}$$ (13)

These steps continue until there are no more changes in cluster assignments. The number of clusters, *k*, was determined by calculating the distance *d* for different values of *k* and using the elbow method to determine the amount of clusters best describing the data.

## Supporting information

S1 Fig Accuracies for several trained agents. The solutions found by the reinforcement learning algorithm could vary quite a bit, due to the variable nature of the learning process, where exploration and exploitation have to be balanced. Exploitation of a certain policy might prevent the agent from learning the best possible solution. To get an idea of the variability in performance we trained several agents. Panel A in S1 Fig shows the accuracy of the agent used for our analyses (black line) together with the accuracies of several other trained agents (gray lines). Panel B in S1 Fig shows the generalizing accuracy of the agent trained on only two noise levels (black line) with the generalizing accuracies of several other agents trained on only two noise levels (gray lines). **(A)** Accuracies for agents trained on all noise levels. The black line shows the original agent used for the analysis in the main paper. The gray lines are the accuracies achieved by agents during several other training sessions. **(B)** Accuracies for agents trained only two noise levels (red dots). The black line shows the original generalizing agent shown in Fig 6. The gray lines show the generalizing accuracies achieved by several other agents trained on these two noise levels.
(TIF)

S2 Fig Trial length distributions for correct and incorrect trials. To compare trial lengths on correct versus incorrect trials we made distributions for both conditions. Since there are few incorrect trials, the data was averaged over bins of 6 time steps to obtain clearer figures. The bin count was normalized by the total number of trials per noise level to make a better comparison, given there were only few incorrect trials. **(A)** Trial length distribution for correct trials. **(B)** Trial length distribution for incorrect trials.
(TIF)

S3 FigPsychophysical kernel.To see how strongly the input at every time step contributed to the final decision between class 1 and class 2 we computed the psychophysical kernel (PK). This is defined as the amplitude of the classification image for every time step, where the classification image is the difference between the mean stimulus preceding choice 1 and the mean stimulus preceding choice 2 [44]. At every time step the psychophysical kernel for stimuli, *s* is given by
$$\begin{array}{c} PK_{t}={\left\langle s_{t}\right\rangle }_{D=1}-{\left\langle s_{t}\right\rangle }_{D=2} \end{array}$$(14)
where 〈*s*_*t*_〉_*D* = 1_ means the average over the stimuli shown at time *t*, for all trials that led to decision *D* = 1. The psychophysical kernel remains doesn’t change over time, so evidence at every time step contributes equally to the final decision. The psychophysical kernel shows the amplitude of the classification image at every time step.(TIF)
