# Supplementary figures and images for: Emergent mechanisms of evidence integration in recurrent neural networks
Source: PLoS One. 2018 Oct 16;13(10):e0205676. doi: 10.1371/journal.pone.0205676 (PMC6191121; doi:10.1371/journal.pone.0205676)

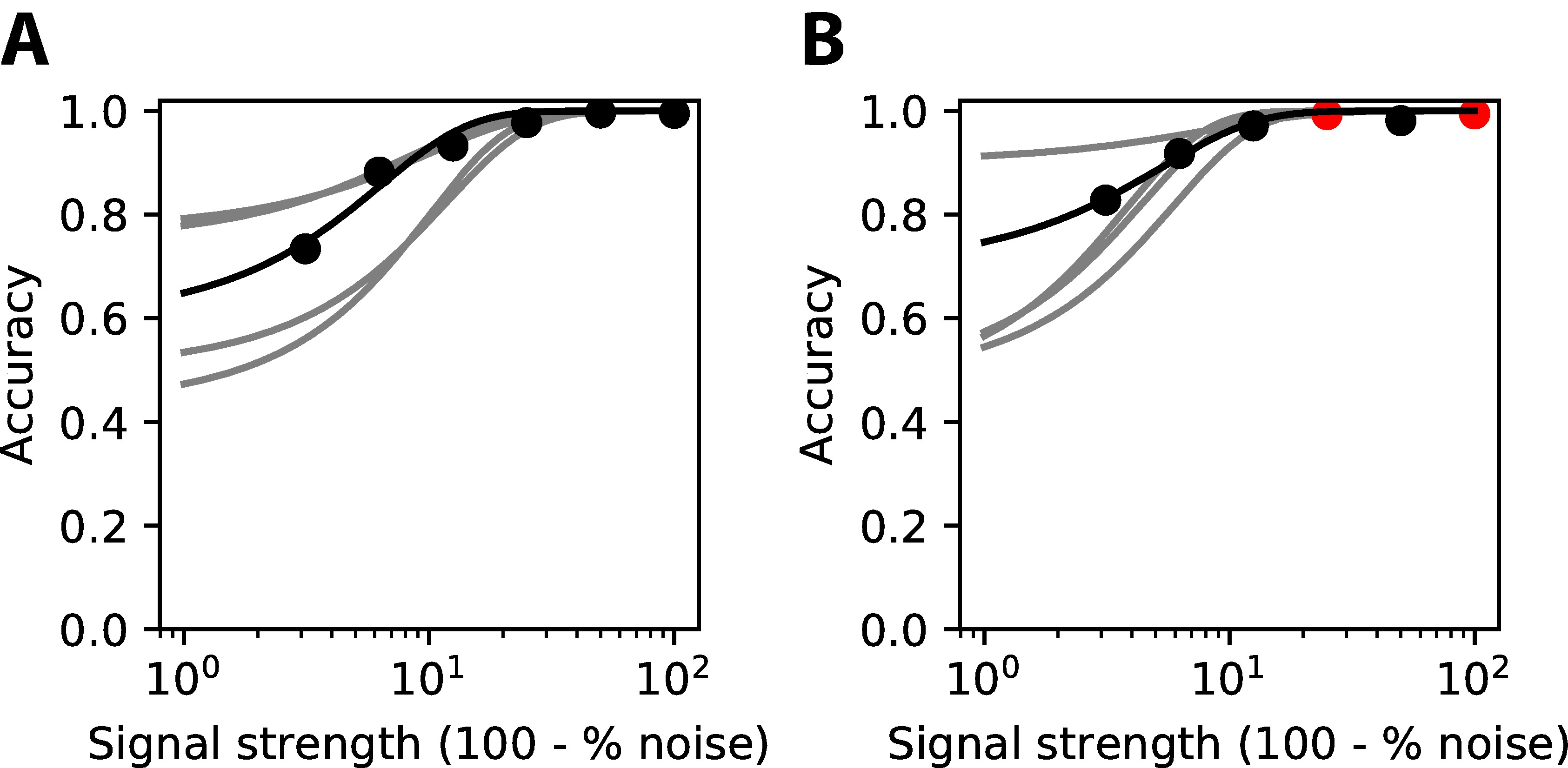

Supplement: S1 Fig — The solutions found by the reinforcement learning algorithm could vary quite a bit, due to the variable nature of the learning process, where exploration and exploitation have to be balanced. Exploitation of a certain policy might prevent the agent from learning the best possible solution. To get an idea of the variability in performance we trained several agents. Panel A in S1 Fig shows the accuracy of the agent used for our analyses (black line) together with the accuracies of several other trained agents (gray lines). Panel B in S1 Fig shows the generalizing accuracy of the agent trained on only two noise levels (black line) with the generalizing accuracies of several other agents trained on only two noise levels (gray lines). (A) Accuracies for agents trained on all noise levels. The black line shows the original agent used for the analysis in the main paper. The gray lines are the accuracies achieved by agents during several other training sessions. (B) Accuracies for agents trained only two noise levels (red dots). The black line shows the original generalizing agent shown in Fig 6. The gray lines show the generalizing accuracies achieved by several other agents trained on these two noise levels. (TIF) [file pone.0205676.s001.tif]

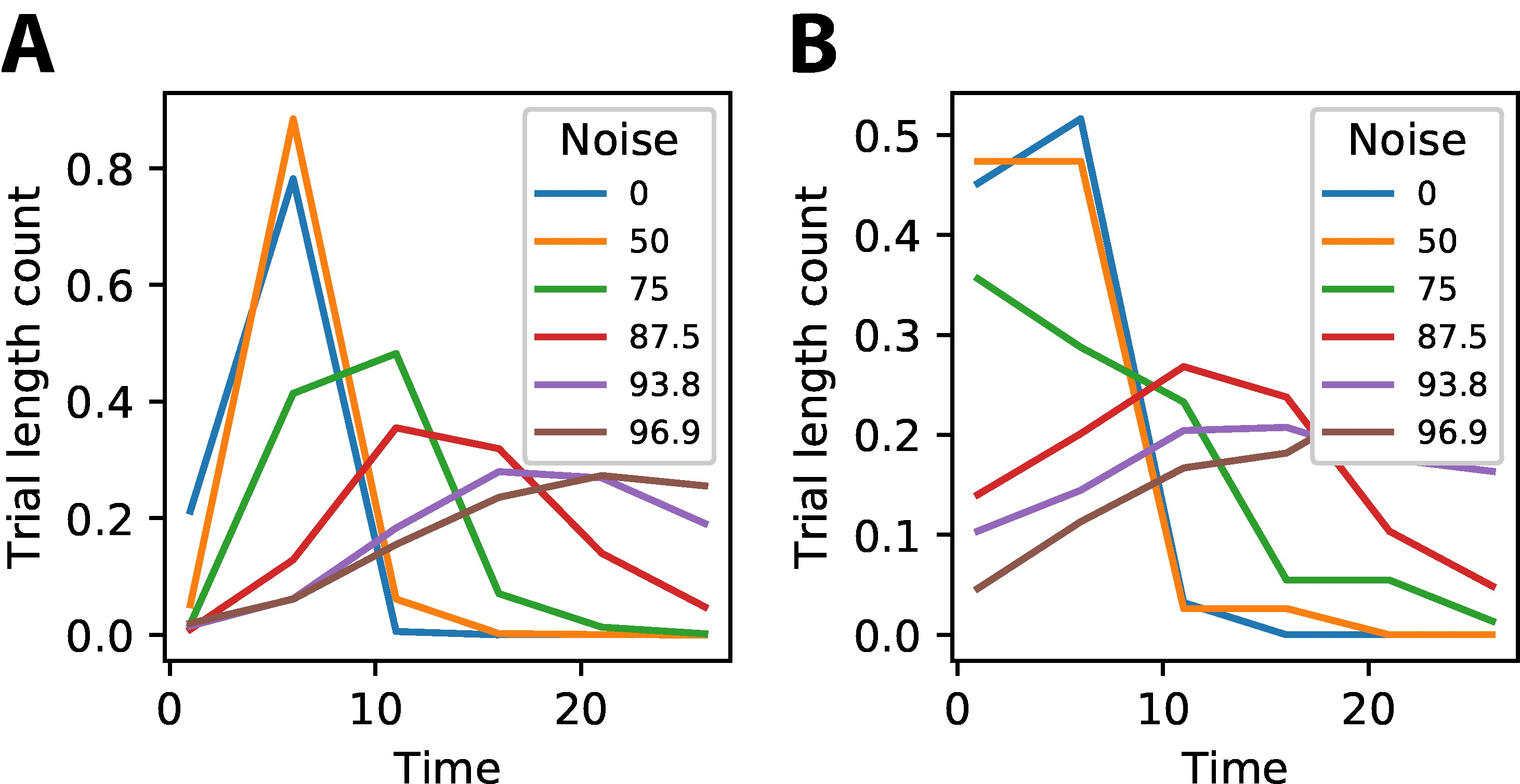

Supplement: S2 Fig — To compare trial lengths on correct versus incorrect trials we made distributions for both conditions. Since there are few incorrect trials, the data was averaged over bins of 6 time steps to obtain clearer figures. The bin count was normalized by the total number of trials per noise level to make a better comparison, given there were only few incorrect trials. (A) Trial length distribution for correct trials. (B) Trial length distribution for incorrect trials. (TIF) [file pone.0205676.s002.tif]

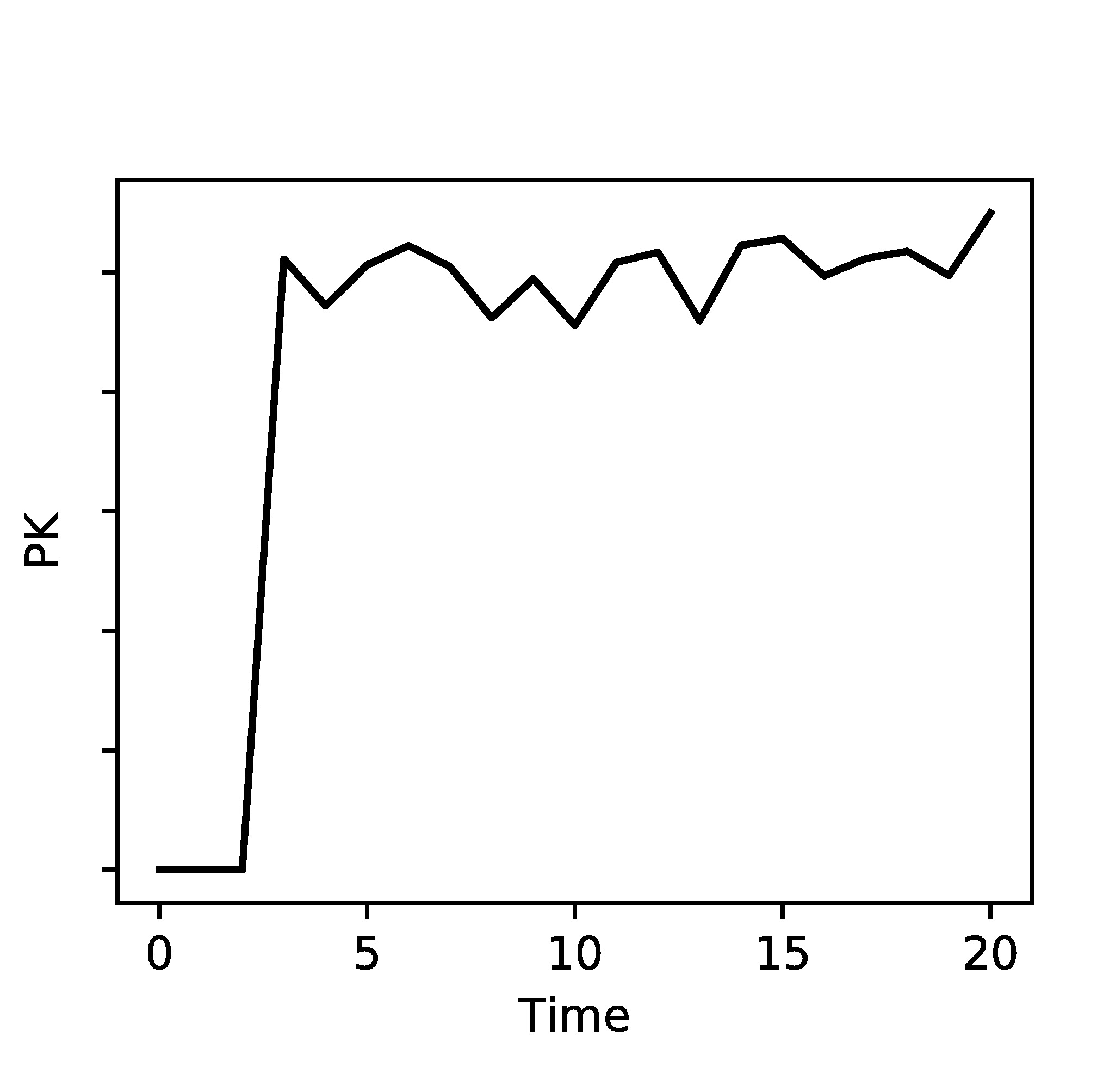

Supplement: S3 Fig — To see how strongly the input at every time step contributed to the final decision between class 1 and class 2 we computed the psychophysical kernel (PK). This is defined as the amplitude of the classification image for every time step, where the classification image is the difference between the mean stimulus preceding choice 1 and the mean stimulus preceding choice 2 [44]. At every time step the psychophysical kernel for stimuli, s is given by PKt=⟨st⟩D=1-⟨st⟩D=2(14) where 〈st〉D = 1 means the average over the stimuli shown at time t, for all trials that led to decision D = 1. The psychophysical kernel remains doesn’t change over time, so evidence at every time step contributes equally to the final decision. The psychophysical kernel shows the amplitude of the classification image at every time step. (TIF) [file pone.0205676.s003.tif]
